# Long non‐coding RNA MALAT1 promotes Th2 differentiation by regulating microRNA‐135b‐5p/GATA‐3 axis in children with allergic rhinitis

**DOI:** 10.1002/kjm2.12587

**Published:** 2022-09-23

**Authors:** Xiong‐Hui Wu, Si‐Jun Zhao, Wei‐Qing Huang, Li‐Hua Huang, Xin‐You Luo, Song‐Liang Long

**Affiliations:** ^1^ Department of Otorhinolaryngology Head and Neck Surgery Hunan Children's Hospital Changsha Hunan People's Republic of China; ^2^ Department of Neonatology Hunan Children's Hospital Changsha Hunan People's Republic of China; ^3^ Laboratory for Medical Center The Third Xiangya Hospital of Central South University Changsha Hunan People's Republic of China

**Keywords:** allergic rhinitis, GATA binding protein 3, long non‐coding RNA MALAT1, microRNA‐135b‐5p, T‐helper 2 differentiation

## Abstract

Allergic rhinitis (AR) threatens patient survival. CD4^+^ T cells play key roles in AR progression. Long non‐coding RNAs (lncRNAs) are key regulators of cell differentiation. Therefore, we investigated the molecular mechanism of the lncRNA metastasis‐associated lung adenocarcinoma transcript 1 (MALAT1) in AR. Expression levels of MALAT1, microRNA (miR)‐135b‐5p, interleukin‐4 (IL‐4), and GATA‐binding protein 3 (GATA‐3) in the nasal mucosa of AR patients were quantified. CD4^+^ T cells were isolated from the peripheral blood of healthy volunteers and treated with ovalbumin (OVA) and Th2 inducers. After MALAT1 and miR‐135b‐5p levels changed in CD4^+^ T cells, the proportion of IL‐4‐expressing cells and the levels of IL‐4 and GATA‐3 in OVA‐induced CD4^+^ T cells were determined. Binding relationships among MALAT1, miR‐135b‐5p, and GATA‐3 were predicted and verified. Rescue experiments were performed to confirm the role of the MALAT1/miR‐135b‐5p/GATA‐3 axis in Th2 differentiation of CD4^+^ T cells. MALAT1, IL‐4, and GATA‐3 expression was upregulated, whereas miR‐135b‐5p expression was downregulated, in patients with AR. MALAT1 knockdown or miR‐135b‐5p overexpression in CD4^+^ T cells notably decreased the proportion of IL‐4‐expressing cells and downregulated GATA‐3 and IL‐4 expression in OVA‐induced CD4^+^ T cells. MALAT1 and GATA‐3 exhibited competitive binding toward miR‐135b‐5p. MALAT1 facilitated CD4^+^ T cell Th2 differentiation via the miR‐135b‐5p/GATA‐3 axis. MALAT1 facilitated AR development by facilitating CD4^+^ T cell Th2 differentiation via the miR‐135b‐5p/GATA‐3 axis. This study may provide guidance for clinical treatment of AR.

## 1 INTRODUCTION

Allergic rhinitis (AR), the most prevalent chronic disease in childhood, notably reduces the quality of life of children.^1^, ^2^ Children with AR may experience sleep disorders, and the severity of AR is correlated with the intensity of sleep disorders.^3^ AR is closely associated with asthma, with asthma representing the main risk factor for AR onset.^2^ The main symptoms of AR include nasal congestion, rhinorrhea, and sneezing.^4^ AR is defined as an immunoglobulin E (IgE)‐mediated inflammatory disease of the nasal wall and is caused by the inhalation of allergens in the environment.^5^, ^6^ Dust mites, pollen, molds, and animals may trigger AR symptoms.^7^ Furthermore, early exposure to antibiotics within the first year of life contributes to AR development in children.^8^ Nevertheless, AR treatment primarily focuses on the use of desensitizing drugs, which only target the symptom.^9^ Therefore, it is essential to explore the underlying mechanism of AR to optimize AR treatment in children.

CD4^+^ T cells, which are crucial components of the immune system, play a role in inflammatory disorders.^10^ Several studies have shown that CD4^+^ T cells play a crucial role in AR.^11^, ^12^ Specifically, CD4^+^ T cells can differentiate into several helper subsets (T‐helper [Th]1, Th2, Th17, regulatory T, and T follicular helper cells) upon activation by pathogens in the microenvironment.^13^, ^14^ AR is characterized by an imbalance of Th1/Th2 differentiation, with a predominance of Th2 differentiation.^15^ Therefore, this study was designed to explore the molecular mechanism of CD4^+^ T cell Th2 differentiation in AR with the aim of developing novel approaches for more effective treatment of AR in children.

Long non‐coding RNAs (lncRNAs) are potent regulators of cell differentiation, response to stimulation, and immune response.^16^ As shown previously, lncRNAs play key roles in AR pathogenesis.^17^ The lncRNA metastasis‐associated lung adenocarcinoma transcript 1 (MALAT1) has been reported to play critical roles in multiple physiological processes.^18^ MALAT1 is also involved in various inflammatory diseases.^19^, ^20^ MALAT1 regulates the Th1/Th2 balance within CD4+ T cells by decoying miR‐155.^21^ Th2 differentiation involves transcription factors such as GATA‐binding protein 3 (GATA‐3), and GATA‐3 can increase the secretion of interleukin‐4 (IL‐4) by Th2 cells.^22^ Additionally, the role of microRNAs (miRNAs) in modulating allergic inflammation in AR is gaining attention.^23^, ^24^ miR‐135b has been shown to be involved in asthma.^25^ Nevertheless, the interplay between MALAT1 and miR‐135b‐5p in AR remains unclear.

Therefore, we speculated that MALAT1 might regulate CD4^+^ T cell Th2 differentiation in AR, depending on miR‐135b‐5p‐mediated GATA‐3. Consequently, we performed a series of histological and molecular experiments to identify the underlying molecular mechanisms of the involvement of MALAT1 in Th2 differentiation in AR to develop novel therapies against AR progression.

## 2 MATERIALS AND METHODS

### 2.1 Clinical samples

Nasal mucosa tissues were scraped from the inferior turbinate surface of 10 children with AR and 10 healthy children without AR using a plastic curette. The diagnostic criteria for AR were as follows: (1) a typical AR history of more than 2 years, (2) a clinical manifestation of runny nose, (3) a positive skin prick test for a specific antigen, and (4) a serum‐specific IgE level of more than 0.3 IU/ml. Information on the subjects is presented in Table 1.

**TABLE 1:** Information on AR patients and healthy volunteers included

	Normal (*n* = 10)	AR (*n* = 10)	*p*
Age (years)	8.80 ± 3.50	10.50 ± 3.10	0.27
Height (cm)	138.80 ± 11.47	140.60 ± 9.25	0.7
Weight (kg)	36.91 ± 4.76	38.16 ± 4.49	0.55
BMI	19.61 ± 4.29	19.54 ± 3.35	0.97
Sex (male/female)	6/4	5/5	>0.99

*Note*: Data were expressed as mean ± standard deviation.

Abbreviations: AR, allergic rhinitis; BMI, body mass index.

### 2.2 CD4 ^+^ T cell isolation and induction for Th2 differentiation

Peripheral blood was collected from healthy volunteers, and peripheral blood mononuclear cells (PBMCs) were obtained by density gradient centrifugation.^26^ CD4^+^ T cells were isolated from PBMCs by magnetic‐activated cell sorting (MACS CD4^+^ T cell Isolation Kit, Miltenyi Biotech), after which CD4^+^ T cells were cultured for 7 days in RPMI 1640 supplemented with anti‐CD3 (3 μg/ml), anti‐CD28 (5 μg/ml), IL‐4 (20 ng/ml), and anti‐IFN‐γ (20 μg/ml) to induce Th2 differentiation. Ovalbumin (OVA) (1 μg/ml) was added to induce an inflammatory response in the CD4^+^ T cells. The culture medium was refreshed every 2–3 days, and the concentrations of the additives were identical in the media. All CD4^+^ T cells were obtained from healthy volunteers.

### 2.3 Cell culture

Isolated CD4^+^ T cells were cultured in RPMI 1640 medium (Sigma–Aldrich). The medium was supplemented with antibiotics (penicillin and streptomycin), glutamine, and fetal bovine serum and replaced every 2–3 days. The cell cultures were incubated at 37°C with 5% CO_2_. The cell concentration was standardized to 1 × 10^6^/ml before the cells were used for the following experiments.

### 2.4 Cell transfection

As described in a previous study,^27^ lentiviral plasmids were generated by HEK293T cells. Briefly, cells were transfected with lentiviral vectors and a packaging mixture. The supernatant of HEK293T cells was collected and the viral concentration was calculated 48 h after transfection, using the fluorescence ratio determined by a flow cytometer.

CD4^+^ T cells (1 × 10^6^/ml) were transfected or co‐transfected with the MALAT1 knockdown lentiviral vector (sh‐MALAT1, 20 μl, 2 × 10^8^ TU/ml virus titer), MALAT1 overexpression vector (LV‐MALAT1, 20 μl, 2 × 10^8^ TU/ml virus titer), miR‐135b‐5p mimic (50 nM), miR‐135b‐5p inhibitor (50 nM), GATA‐3 overexpression lentiviral vector (LV‐GATA‐3, 20 μl, 2 × 10^8^ TU/ml virus titer), GATA‐3 knockdown vector (sh‐GATA‐3, 20 μl, 2 × 10^8^ TU/ml virus titer), and their negative controls (LV‐NC, sh‐NC, inhibitor NC, and mimic NC). All lentiviral vectors were supplied by GenePharma Co., Ltd. Transfection of the miR‐135b‐5p mimic and miR‐135b‐5p inhibitor was performed using LipoFiter™ transfection reagent (Hanbio Biotechnology Co., Ltd.) according to the manufacturer's instructions. Three replicates were used for the transfection. After transfection for 24 h, CD4^+^ T cells were induced to differentiate into Th2 cells.

### 2.5 Quantitative reverse transcription‐polymerase chain reaction

Total RNA was extracted from the cells or tissues in each group using TRIzol reagent (Invitrogen Inc.). The concentration and purity of the RNA samples were determined. Qualified RNA was adjusted to an appropriate concentration and subjected to reverse transcription using a reverse transcription kit (Takara) and random primers according to the corresponding instructions. A LightCycler 480 fluorescence quantitative PCR instrument (Roche Diagnostics) was used to detect gene expression. The reaction conditions complied with the instructions for SYBR Green M (Roche Diagnostics): 5 min pre‐denaturation (95°C), followed by 40 cycles of 10 s denaturation (95°C), 10 s annealing (60°C), and 20 s extension (72°C). Three replicates were used for each reaction. U6 served as the internal reference for miR, and glyceraldehyde‐3‐phosphate dehydrogenase (GAPDH) was used as the internal reference for mRNA and lncRNA. Data were analyzed using the 2^−ΔΔCT^ method. The primer sequences used are listed in Table 2.

**TABLE 2:** Primer sequence for qRT‐PCR

Name of primer	Sequences
*U6*‐F	CTCGCTTCGGCAGCACAT
*U6*‐R	AACGCTTCACGAATTTGCGT
Hsa‐miR‐135b‐5p‐F	AGCTATGGCTTTTCATTCCTATG
Hsa‐miR‐135b‐5p‐R	CTCAACTGGTGTCGTGGAGTC
*GAPDH*‐F	CGGACCAATACGACCAAATCCG
*GAPDH*‐R	AGCCACATCGCTCAGACACC
MALAT1‐F	GTGATGCGAGTTGTTCTCCG
MALAT1‐R	CTGGCTGCCTCAATGCCTAC
GATA‐3‐F	TGTCTGCAGCCAGGAGAGC
GATA‐3‐R	ATGCATCAAACAACTGTGGCCA
IL‐4‐F	ATCTTTGCTGCCTCCAAGAACA
IL‐4‐R	CTCTGGTTGGCTTCCTTCACA

Abbreviations: F, forward; qRT‐PCR, quantitative reverse transcription‐polymerase chain reaction; R, reverse.

### 2.6 Western blotting

Cells and tissues from each group were lysed using RIPA lysis buffer (Beyotime Biotechnology) and centrifuged to obtain protein samples. GAPDH was used as the internal reference. The concentration of each protein sample was measured using a bicinchoninic acid kit (Beyotime) to ensure equal amounts of each sample. An appropriate volume of proteins was added and mixed with a loading buffer (Beyotime), which was then heated in a boiling water bath for 3 min to denature the protein. A 10% sodium dodecyl sulfate‐polyacrylamide gel electrophoresis (SDS‐PAGE) gel (configured as instructed by the SDS‐PAGE gel preparation kit [Beyotime]) was used for protein separation. Proteins underwent 1–2 h of electrophoresis; the voltage was changed from 80 to 120 V after bromophenol blue reached the gel. Following protein transfer to membranes in an ice bath at 300 mA for 60 min, the membranes were rinsed in the washing solution for 1–2 min and then sealed in the sealing solution at room temperature for 60 min or at 4°C overnight. Next, the membranes were incubated for 1 h with rabbit anti‐human primary antibodies GAPDH (5174 s, 1:1000, Cell Signaling) and antibody against GATA‐3 (ab199428, 1:1000, Abcam) on a shaking table at room temperature, followed by three washes with washing solution for 10 min each. Next, the secondary antibody, horseradish peroxidase‐labeled goat anti‐rabbit immunoglobulin G (IgG, 1:5000, CoWin Biosciences), was added for 1 h of incubation at room temperature. After three washes with washing solution for 10 min each, the membranes were dripped using a developer. A chemiluminescence imaging system (Bio‐Rad Inc.) was used for detection. Each experiment was independently repeated thrice.

### 2.7 Dual‐luciferase reporter gene assay

Using starBase (http://starbase.sysu.edu.cn/agoClipRNA.php?source=lncRNA) and starBase (http://starbase.sysu.edu.cn/agoClipRNA.php?source=mRNA), the binding site between MALAT1 and miR‐135b‐5p, as well as that between miR‐135b‐5p and GATA‐3, was predicted. Based on the predicted results, wild‐type (wt) and mutant (mut) sequences of the binding sites (mut‐MALAT1, wt‐MALAT1, mut‐GATA‐3, and wt‐GATA‐3) were designed, synthesized, and inserted into the luciferase reporter gene vector (pGL3‐promoter, Promega). The vectors were then co‐transfected with the miR‐135b‐5p mimic (50 nM, GenePharma) (mimic + mut‐MALAT1, mimic + wt‐MALAT1, mimic + mut‐GATA‐3, mimic + wt‐GATA‐3) or miR‐135b‐5p mimic NC (50 nM, GenePharma) (mimic NC + mut‐MALAT1, mimic NC + wt‐MALAT1, mimic NC + mut‐GATA‐3, and mimic NC + wt‐GATA‐3) into HEK293T cells. After transfection, the fluorescence intensity of each group was detected using a dual‐luciferase reporter gene detection kit (Promega) to determine miR‐135b‐5p binding with MALAT1 and GATA‐3. Each experiment was independently repeated thrice.

### 2.8 RNA pull‐down

RNA pull‐down assays were performed using the Pierce™ Magnetic RNA‐Protein Pull‐Down Kit (Millipore). Biotinylated MALAT1, biotinylated GATA‐3 (Geneseed Biotech Co., Ltd.), and biotinylated NC were incubated (25°C, 2 h) with HEK293T cell lysates. Next, MALAT1/miR‐135b‐5p or GATA‐3/miR‐135b‐5p complexes were captured using streptavidin‐labeled immunomagnetic beads at 25°C for 1 h and then incubated with proteinase K‐containing buffer for 1 h at 25°C. The eluted complexes were detected by quantitative reverse transcription‐polymerase chain reaction (qRT‐PCR).

### 2.9 Identification of IL‐4‐expressing cells

The percentage of IL‐4‐expressing cells was determined using flow cytometry. Briefly, sorted CD4^+^ T cells (1 × 10^6^/ml) were fixed in 2% paraformaldehyde at room temperature for 2 h, followed by 30 min of incubation with 0.5% saponins to increase cell membrane permeability. After washing with PBS, the cells were incubated with fluorescein isothiocyanate‐labeled antibody (anti‐IL‐4) for 1 h. A FACSCanto II flow cytometer (BD Biosciences) was used to analyze the stained cells. Each experiment was independently repeated thrice.

### 2.10 Enzyme‐linked immunosorbent assay

IL‐4 levels in the cell culture supernatant were quantitatively analyzed using quantitative ELISA kits (R&D Systems), according to the manufacturer's instructions. Each experiment was independently repeated thrice.

### 2.11 Statistical analysis

GraphPad software (version 7.0) was used for data analysis. All data are presented as the mean ± standard deviation ($\bar{x}$ ± s). *T* test was utilized for analysis of comparison between two groups. One‐way analysis of variance (ANOVA) was used to compare multiple groups, followed by Tukey's multiple comparison test. Statistical significance was set at *p* < 0.05.

## 3 RESULTS

### 3.1 MALAT1 and GATA‐3 expression was upregulated in allergic rhinitis patients

**FIGURE 1:**
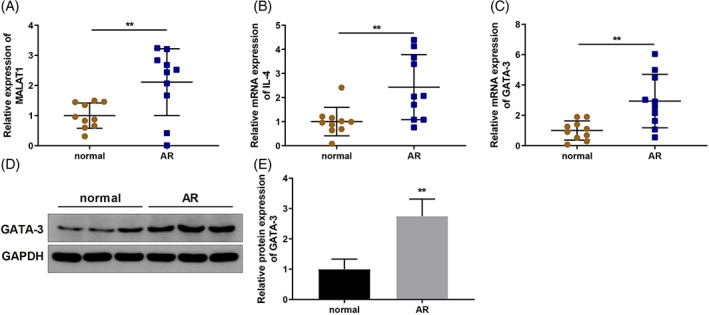
MALAT1 and GATA‐3 expression levels are upregulated in AR patients. Nasal mucosa tissue samples of 10 AR patients and 10 normal volunteers were collected. (A) MALAT1 expression in nasal mucosal tissues was quantified via qRT‐PCR; (B) IL‐4 expression in nasal mucosal tissues was quantified via qRT‐PCR; (C‐E) GATA‐3 expression in nasal mucosal tissues was quantified via qRT‐PCR (C) and WB (D‐E). *N* = 10, **p < 0.01, compared to the normal group. AR, allergic rhinitis; GATA‐3, GATA‐binding protein 3; MALAT1, metastasis‐associated lung adenocarcinoma transcript 1; qRT‐PCR, quantitative reverse transcription‐polymerase chain reaction; WB, western blotting

AR is characterized by an imbalance in Th1/Th2 differentiation; under this pathological condition, Th2 differentiation increases.^15^ The lncRNA MALAT1 has been implicated in the regulation of Th1/Th2 balance in CD4^+^ T cells.^21^ Th2 differentiation is dependent on transcription factors such as GATA‐3, and GATA‐3 can lead to IL‐4 production.^22^ Therefore, we presumed that MALAT1 might regulate AR progression by regulating GATA‐3 expression. Nasal mucosa tissue samples from 10 AR patients and 10 normal volunteers were collected, and MALAT1 and IL‐4 expression levels were determined using qRT‐PCR. The results indicated that MALAT1 expression was notably elevated (Figure 1A) (*p* < 0.01) and that IL‐4 expression was abnormally upregulated in AR patients (Figure 1B) (*p* < 0.01). GATA‐3 expression was quantified via qRT‐PCR and WB and exhibited an obvious upregulation in AR patients (Figure 1C‐E) (*p* < 0.01). These data indicate that MALAT1 and GATA‐3 levels were upregulated in patients with AR.

### 3.2 MALAT1 and GATA‐3 competitively bound to miR‐135b‐5p

**FIGURE 2:**
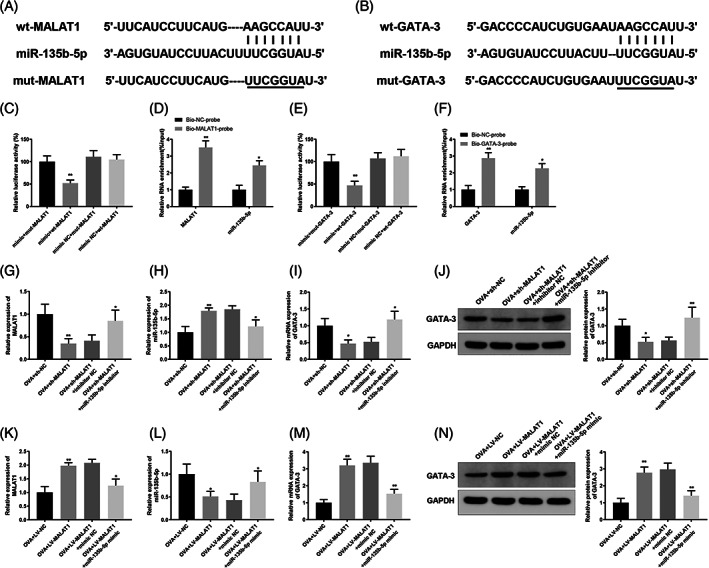
MALAT1 competes with GATA‐3 to bind to miR‐135b‐5p. (A) Binding site between miR‐135b‐5p and MALAT1 3'UTR; (B) binding site between miR‐135b‐5p and GATA‐3 3'UTR; (C) dual‐luciferase reporter gene assay was used to confirm the interaction between MALAT1 and miR‐135b‐5p, **p < 0.01, compared to the OVA + mimic NC + wt‐MALAT1 group; (D) RNA pull‐down assay was used to verify the interaction between MALAT1 and miR‐135b‐5p, *p < 0.05, **p < 0.01, compared to the bio‐NC‐probe group; (E) binding between miR‐135b‐5p and GATA‐3 was verified using dual luciferase reporter gene assay, **p < 0.01, compared to the OVA + mimic NC + wt‐GATA‐3 group; (F) RNA pull‐down assay was used to verify the interaction between miR‐135b‐5p and GATA‐3, *p < 0.05, **p < 0.01, compared to the bio‐NC‐probe group. CD4^+^ T cells were transfected with sh‐MALAT1 or LV‐MALAT1 lentiviral vectors, sh‐MALAT1 plus miR‐135b‐5p inhibitor, or LV‐MALAT1 plus miR‐135b‐5p mimic and then induced with OVA and Th2 inducers. (G–N) qRT‐PCR was used to quantify MALAT1 (G, K), miR‐135b‐5p (H, L) or GATA‐3 (I, M) expression levels, and WB was utilized to quantify GATA‐3 expression levels (J, N) in CD4^+^ T cells, *p < 0.05, **p < 0.01, compared to the OVA + sh‐NC/OVA + LV‐NC group or OVA + sh‐MALAT1 + inhibitor NC/OVA + LV‐MALAT1 + mimic NC group. Each experiment was independently repeated three times. AR, allergic rhinitis; GATA‐3, GATA‐binding protein 3; IL, interleukin; lncRNA, long non‐coding RNA; MALAT1, metastasis‐associated lung adenocarcinoma transcript 1; miR, microRNA; OVA, ovalbumin; qRT‐PCR, quantitative reverse transcription‐polymerase chain reaction; WB, western blotting

Since we found upregulated MALAT1 and GATA‐3 in AR tissues, we postulated that MALAT1 might act as a competitive endogenous RNA in AR. The online database starBase (http://starbase.sysu.edu.cn/agoClipRNA.php?source=lncRNA) predicted that both MALAT1 and GATA‐3 have a binding site within miR‐135b‐5p. Therefore, wt and mut plasmids containing MALAT1/GATA‐3 3′UTR (wt‐MALAT1, mut‐MALAT1, wt‐GATA‐3, and mut‐GATA‐3) were constructed to perform dual‐luciferase reporter gene assays (Figure 2A,B). Luciferase activity was noticeably reduced in the mimic + wt‐MALAT1 group (Figure 2C) (*p* < 0.01). Based on RNA pull‐down analysis, endogenous miR‐135b‐5p was pulled down using the biotinylated MALAT1 probe (Figure 2D) (*p* < 0.05). Moreover, a luciferase reporter assay for the binding between miR‐135b‐5p and GATA‐3 revealed that the mimic + wt‐GATA‐3 group exhibited dramatically reduced luciferase activity (Figure 2E) (*p* < 0.01), and RNA pull‐down analysis revealed that endogenous miR‐135b‐5p was pulled down by biotinylated GATA‐3 probes (Figure 2F) (*p* < 0.05). CD4^+^ T cells were transfected with sh‐MALAT1 or LV‐MALAT1 lentiviral vectors, sh‐MALAT1 plus miR‐135b‐5p inhibitor, or LV‐MALAT1 plus miR‐135b‐5p mimic and then exposed to OVA and Th2 inducers. qRT‐PCR and WB results revealed that sh‐MALAT1 transfection before OVA induction remarkably downregulated MALAT1 and GATA‐3 levels and upregulated miR‐135b‐5p in CD4^+^ T cells, while MALAT1 and GATA‐3 levels increased and miR‐135b‐5p levels decreased after co‐transfection with miR‐135b‐5p inhibitor (Figure 2G–J) (*p* < 0.05). LV‐MALAT1 transfection caused elevated MALAT1 and GATA‐3 levels and reduced miR‐135b‐5p expression level, but the effect of co‐transfection with miR‐135b‐5p mimic on the expression of these genes followed the opposite trend (Figure 2K–N) (*p* < 0.05). These data suggest that MALAT1 competes with GATA‐3 for binding to miR‐135b‐5p.

### 3.3 MALAT1 knockdown or miR‐135b‐5p overexpression inhibited ovalbumin‐induced CD4 ^+^ T cell Th2 differentiation

**FIGURE 3:**
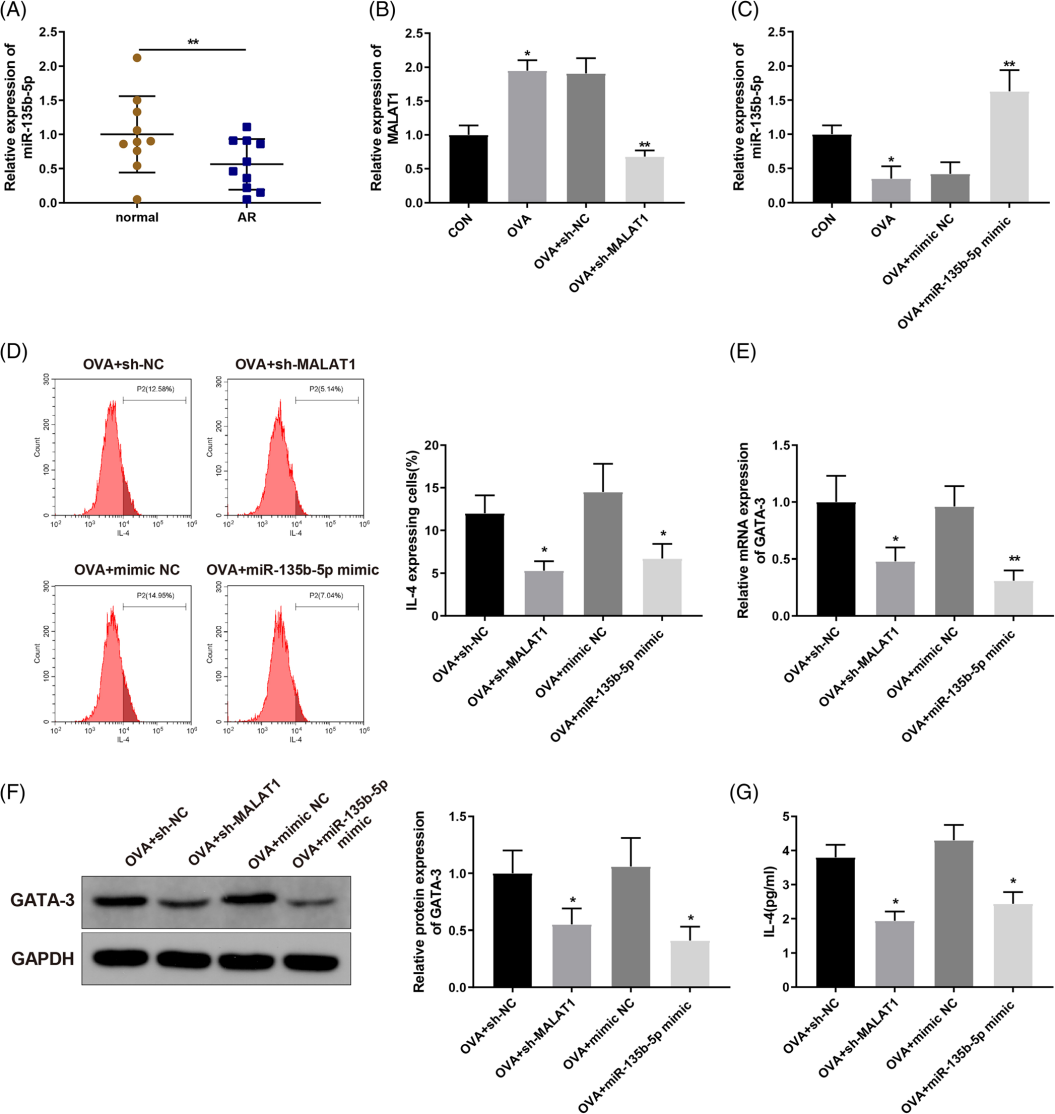
MALAT1 knockdown or miR‐135b‐5p overexpression inhibits OVA‐induced Th2 differentiation of CD4^+^ T cells. (A) miR‐135b‐5p expression levels in the collected nasal mucosa tissue samples were quantified via qRT‐PCR and compared to normal group. CD4^+^ T cells were transfected with sh‐MALAT1 lentiviral vector or miR‐135b‐5p mimic and induced with OVA and Th2 inducers. (B) qRT‐PCR was performed to quantify MALAT1 expression levels in OVA‐induced CD4^+^ T cells, compared to CON group or OVA + sh‐NC/OVA + mimic NC group; (C) qRT‐PCR was used to quantify miR‐135b‐5p expression levels in OVA‐induced CD4^+^ T cells; (D) Flow cytometry was used to determine the percentage of IL‐4‐expressing cells; (E, F) qRT‐PCR (E) and WB (F) were used to quantify GATA‐3 expression levels in OVA‐induced CD4^+^ T cells; (G) ELISA was used to quantify IL‐4 levels in the supernatant of OVA‐induced CD4^+^ T cells. *p < 0.05, **p < 0.01, compared to the OVA + sh‐NC group or the OVA + mimic NC group. Each experiment was independently repeated three times. AR, allergic rhinitis; ELISA, enzyme‐linked immunosorbent assay; lncRNA, long non‐coding RNA; MALAT1, metastasis‐associated lung adenocarcinoma transcript 1; miR, microRNA; IL, interleukin; GATA‐3, GATA‐binding protein 3; OVA, ovalbumin; qRT‐PCR, quantitative reverse transcription‐polymerase chain reaction; WB, western blotting

To further ascertain whether miR‐135b‐5p plays a role in AR, we examined miR‐135b‐5p expression in the collected AR and normal tissues using qRT‐PCR, and miR‐135b‐5p expression was downregulated in AR tissues (Figure 3A) (*p* < 0.01). CD4^+^ T cells were transfected with sh‐MALAT1 lentiviral vectors or miR‐135b‐5p mimic before exposure to OVA and Th2 inducer. After transfection, MALAT1 and miR‐135b‐5p expression levels in OVA‐induced CD4^+^ T cells were determined using qRT‐PCR. OVA induction substantially potentiated MALAT1 expression in CD4^+^ T cells (Figure 3B) (*p* < 0.05), while MALAT1 expression was notably decreased after transfection with sh‐MALAT1 (Figure 3B) (*p* < 0.01). In contrast, miR‐135b‐5p expression decreased in OVA‐induced CD4^+^ T cells (Figure 3C) (*p* < 0.05), but miR‐135b‐5p mimic transfection restored its expression (Figure 3C) (*p* < 0.01). The percentage of IL‐4‐expressing cells was determined using flow cytometry. The results revealed that sh‐MALAT1 and miR‐135b‐5p mimic transfection dramatically decreased the percentage of IL‐4‐expressing cells (Figure 3D) (*p* < 0.05). In addition, GATA‐3 expression decreased in OVA‐induced CD4^+^ T cells transfected with sh‐MALAT1 or miR‐135b‐5p mimics (Figure 3E,F) (*p* < 0.05). Furthermore, enzyme‐linked immunosorbent assay (ELISA) results indicated that MALAT1 knockdown or miR‐135b‐5p overexpression effectively reduced IL‐4 levels in the supernatant of OVA‐induced CD4^+^ T cells (Figure 3G) (*p* < 0.05).

### 3.4 MALAT1‐facilitated CD4 ^+^ T cell Th2 differentiation through the miR‐135b‐5p/GATA‐3 axis

**FIGURE 4:**
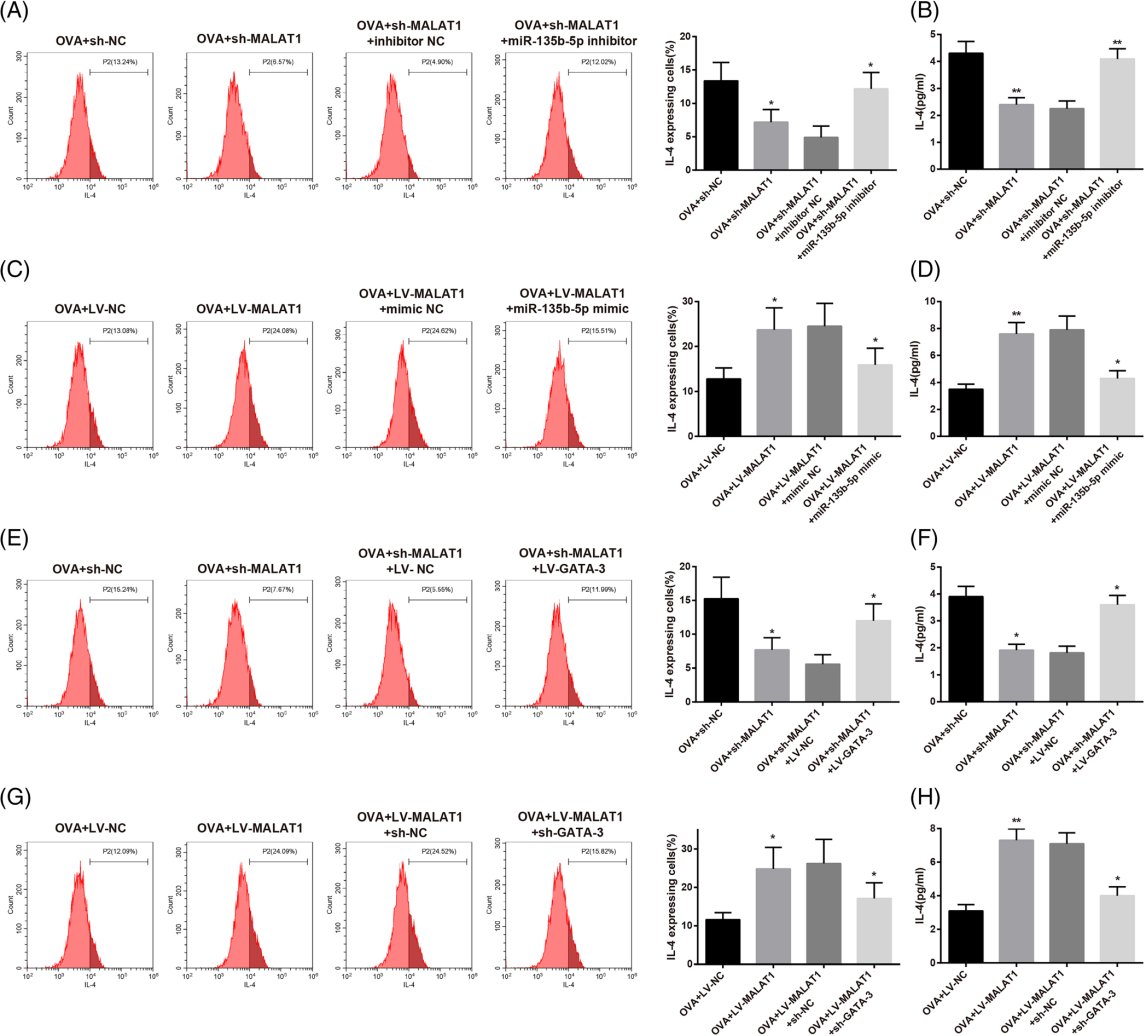
MALAT1 promotes OVA‐induced Th2 differentiation of CD4^+^ T cells via the miR‐135b‐5p/GATA‐3 axis. CD4^+^ T cells were transfected with sh‐MALAT1 lentiviral vector or co‐transfected with sh‐MALAT1 lentiviral vector and miR‐135b‐5p inhibitor, followed by the induction using OVA and Th2 inducers. (A) Percentage of IL‐4‐expressing cells was determined using flow cytometry. (B) IL‐4 level in the supernatant of OVA‐induced CD4^+^ T cells was measured using ELISA, *p < 0.05, **p < 0.01, compared to the OVA + sh‐NC group or the OVA + sh‐MALAT1 + inhibitor NC group. CD4^+^ T cells were transfected with LV‐MALAT1 or co‐transfected with LV‐MALAT1 and miR‐135b‐5p mimic and induced for Th2 differentiation; (C) percentage of IL‐4‐expressing cells was determined using flow cytometry; (D) IL‐4 level in the supernatant of OVA‐induced CD4^+^ T cells was measured using ELISA, *p < 0.05, **p < 0.01, compared to the OVA + LV‐NC group or the OVA + LV‐MALAT1 + mimic NC group. CD4^+^ T cells were transfected with sh‐MALAT1 or co‐transfected with sh‐MALAT1 and LV‐GATA‐3 and induced using OVA and Th2 inducers; (E) Percentage of IL‐4‐expressing cells was determined using flow cytometry; (F) IL‐4 level in the supernatant of OVA‐induced CD4^+^ T cells was measured using ELISA, *p < 0.05, compared to the OVA + sh‐NC group or the OVA + sh‐MALAT1 + LV‐NC group. CD4^+^ T cells were transfected with LV‐MALAT1 or co‐transfected with LV‐MALAT1 and sh‐GATA‐3 and induced using OVA and Th2 inducers. (G) Percentage of IL‐4‐expressing cells was determined using flow cytometry; (H) IL‐4 level in the supernatant of OVA‐induced CD4^+^ T cells was measured using ELISA, *p < 0.05, **p < 0.01, compared to the OVA + LV‐NC group or the OVA + LV‐MALAT1 + sh‐NC group. Each experiment was independently repeated three times. AR, allergic rhinitis; ELISA, enzyme‐linked immunosorbent assay; GATA‐3, GATA‐binding protein 3; IL, interleukin; lncRNA, long non‐coding RNA; MALAT1, metastasis‐associated lung adenocarcinoma transcript 1; miR, microRNA; OVA, ovalbumin; qRT‐PCR, quantitative reverse transcription‐polymerase chain reaction; WB, western blotting

After transfection with sh‐MALAT1 lentiviral vector or co‐transfection of sh‐MALAT1 lentivirus vector and miR‐135b‐5p inhibitor, CD4^+^ T cells were induced for Th2 differentiation. Flow cytometry results revealed that sh‐MALAT1 transfection led to a remarkable decrease in the percentage of IL‐4‐expressing cells and IL‐4 levels in the supernatant of OVA‐induced CD4^+^ T cells (Figure 4A,B) (*p* < 0.05), but co‐transfection with miR‐135b‐5p inhibitor resulted in opposite trends (Figure 4A,B) (*p* < 0.05). Furthermore, MALAT1 overexpression in CD4^+^ T cells increased the percentage of IL‐4‐expressing cells and IL‐4 levels (Figure 4C,D) (*p* < 0.05), which was reversed after co‐transfection with the miR‐135b‐5p mimic (Figure 4C,D) (*p* < 0.05). Moreover, the results showed that co‐transfection with LV‐GATA‐3 rescued the effect of MALAT1 knockdown by decreasing the percentage of IL‐4‐expressing cells and IL‐4 levels in CD4^+^ T cells (Figure 4E,F) (*p* < 0.05). GATA‐3 knockdown effectively weakened the impact of MALAT1 overexpression on the percentage of IL‐4‐expressing cells and IL‐4 levels in CD4^+^ T cells (Figure 4G,H) (*p* < 0.05). Therefore, MALAT1 promoted OVA‐induced Th2 differentiation of CD4^+^ T cells via the miR‐135b‐5p/GATA‐3 axis.

## 4 DISCUSSION

**FIGURE 5:**
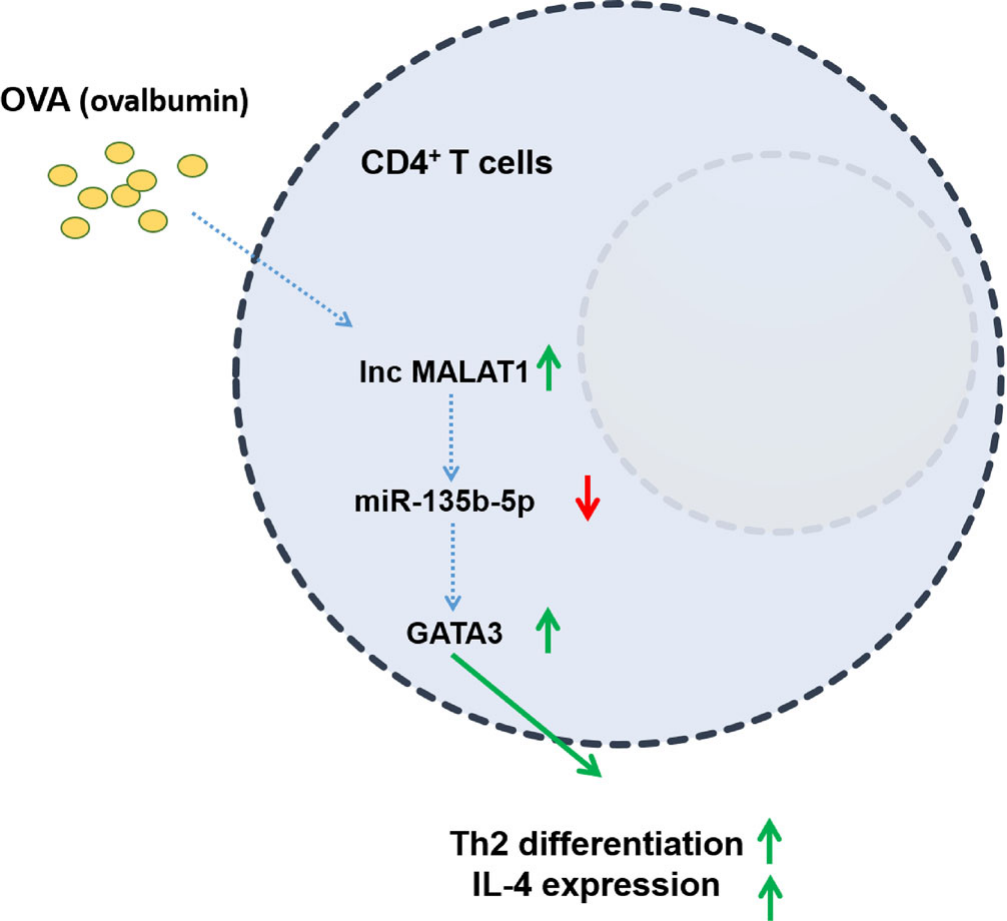
A schematic diagram of long noncoding RNA MALAT1 promoting ovalbumin‐induced Th2 differentiation and IL‐4 expression in CD4^+^ T cells by the miR‐135b‐5p/GATA‐3 axis. Ovalbumin induction upregulated lncRNA MALAT1 expression and downregulated miR‐135b‐5p expression, which upregulated GATA‐3 expression, thereby promoting Th2 differentiation and IL‐4 expression in CD4^+^ T cells. AR, allergic rhinitis; GATA‐3, GATA‐binding protein 3; IL, interleukin; lncRNA, long non‐coding RNA; MALAT1, metastasis‐associated lung adenocarcinoma transcript 1; miR, microRNA; OVA, ovalbumin

AR is a common disease that occurs frequently in children and adolescents.^7^ CD4^+^ T cell Th2 differentiation is intrinsically associated with AR occurrence.^15^ In the present study, we found that lncRNA MALAT1 promoted CD4^+^ T cell Th2 differentiation to facilitate AR development by regulating the miR‐135b‐5p/GATA‐3 axis (Figure 5).

Extensive evidence has validated that MALAT1 is closely associated with inflammation‐associated diseases.^28^, ^29^ miR‐135b‐5p has been found to be poorly expressed in different diseases.^30^, ^31^ The results of this study revealed that MALAT1 expression was notably elevated and miR‐135b‐5p expression decreased in patients with AR. In support of these results, MALAT1 has been shown to exhibit a close relationship with chronic rhinosinusitis.^32^ MALAT1 is overexpressed and miR‐135b is sparsely expressed in asthmatic children.^21^, ^25^

Among the subsets of CD4^+^ T cells, Th2 cells have shown a prominent effect on allergy‐associated inflammatory pathologies, such as AR.^12^ Differentiation into Th2 cells is intrinsically related to transcription factors, such as GATA‐3 (which promotes Th2 cells to secrete IL‐4).^22^ IL‐4 upregulation was previously considered to be positively correlated with AR severity.^33^, ^34^ In this study, IL‐4 and GATA‐3 expressions were abnormally upregulated in patients with AR. Consistently, a previous study has shown that GATA‐3 regulates Th2 cell differentiation and induces secretion of the Th2 cytokine IL‐4.^35^ Elevated IL‐4 levels promote Th2 cell differentiation.^36^ Next, sh‐MALAT1 lentivirus vector or miR‐135b‐5p mimic was transfected into CD4^+^ T cells, which were then treated with OVA and Th2 inducer to explore the specific effects of MALAT1 and miR‐135b‐5p on AR. Transfection of sh‐MALAT1 lentivirus vector or miR‐135b‐5p mimic dramatically decreased the number of IL‐4‐expressing cells and IL‐4 levels in OVA‐induced CD4^+^ T cells, in addition to reducing GATA‐3 expression levels. Several studies have provided evidence for the above findings. For example, MALAT1 promotes Th2 differentiation within CD4^+^ T cells, which may help develop methods for relieving asthmatic inflammation.^21^ miR‐135b overexpression reduces IL‐4 levels, which is promising for alleviating airway inflammation in asthma.^25^ The above data demonstrated that MALAT1 knockdown or miR‐135b‐5p overexpression inhibited OVA‐induced Th2 differentiation of CD4^+^ T cells.

Subsequently, the underlying mechanism by which MALAT1 and miR‐135b‐5p influence CD4^+^ T cell Th2 differentiation in AR was explored. It is well established that lncRNAs can interact with miRNAs to affect the immune system.^16^ Moreover, starBase predicted a binding site between MALAT1 and miR‐135b‐5p. Following a series of experiments, it was identified that MALAT1 targeted miR‐135b‐5p and that miR‐135b‐5p overexpression effectively weakened the effect of MALAT1 on IL‐4 levels in CD4^+^ T cells. As shown previously, hsa‐miR‐135a‐5p has a negative correlation with lncRNA MALAT1 in hypertension.^37^ However, the interactions between MALAT1 and miR‐135b‐5p in AR need to be elucidated, which demonstrates the novelty of the present study. However, mounting evidence shows that the transcription factor GATA‐3 plays a key role in maintaining Th2 cell identity, exhibiting great significance in AR pathogenesis.^38^, ^39^ Therefore, a relationship between GATA‐3 and miR‐135b‐5p was predicted, and GATA‐3 was found to be negatively correlated with miR‐135b‐5p. Consistently, in a previous study, miR‐135b was shown to play an inhibitory role on the Th2 master transcription factor GATA‐3.^40^ Furthermore, suppression of miR‐135b‐5p rescued GATA‐3 downregulation caused by MALAT1 knockdown. GATA‐3 knockdown interfered with the effect of MALAT1 overexpression on IL‐4 levels in CD4^+^ T cells. Similarly, it has been reported that MALAT1 sponges a miRNA and raises GATA‐3 expression to promote Th2 differentiation in CD4^+^ T cells during asthmatic inflammation.^21^ Therefore, MALAT1 competitively binds to miR‐135b‐5p and promotes Th2 differentiation of CD4^+^ T cells by regulating the miR‐135b‐5p/GATA‐3 axis.

Overall, this study suggests that MALAT1 promotes Th2 differentiation within CD4^+^ T cells by sponging miR‐135b‐5p to upregulate GATA‐3, thereby facilitating AR progression. These results provide a possible explanation for AR development from the perspective of molecular mechanisms and demonstrate that MALAT1 can potentially be used as a predictor for the occurrence of AR. Given the regulatory effects of the MALAT1/miR‐135b‐5p/GATA‐3 axis on CD4^+^ T cell Th2 differentiation, an important event intrinsically related to AR occurrence, this study revealed novel potential targets for molecule‐based therapeutic approaches against AR. Although the findings of the current study provide a new perspective for the treatment of AR, their clinical applicability requires further verification.

## CONFLICT OF INTEREST

All authors declare no conflict of interest.

## ETHICAL APPROVAL

This study got approval from the Ethics Committee of Hunan Children's Hospital. All the subjects signed informed consents.
